# Cardiac Arrest Associated With Psilocybin Use and Hereditary Hemochromatosis

**DOI:** 10.7759/cureus.38669

**Published:** 2023-05-07

**Authors:** Suhwoo Bae, Michael Vaysblat, Edward Bae, Ilja Dejanovic, Matthew Pierce

**Affiliations:** 1 Internal Medicine, Donald and Barbara Zucker School of Medicine at Hofstra/Northwell, Manhasset, USA; 2 Medicine, North Shore University Hospital, Brooklyn, USA; 3 Internal Medicine, State University of New York (SUNY) Downstate College of Medicine, Brooklyn, USA; 4 Cardiology, Donald and Barbara Zucker School of Medicine at Hofstra/Northwell, Manhasset, USA; 5 Cardiology, Northwell Health, Manhasset, USA

**Keywords:** substance recreational use, iron overload cardiomyopathy, psilocybin, out-of-hospital cardiac arrest, cardiac hemochromatosis

## Abstract

Recreational drug use is a significant public health concern in various countries. It is well understood that usage of psychedelics/hallucinogens, such as lysergic acid diethylamide (LSD), ecstasy, phencyclidine (PCP), and psilocybin-containing mushrooms, has increased significantly over the last few decades, particularly in adolescents and young adults, yet the effects of these recreational drugs are poorly understood. Psilocybin has recently been studied as an alternative to traditional antidepressant therapies with potentially benign side effects. Here, we present the case of a 48-year-old male with a past medical history of attention-deficit/hyperactivity disorder on lisdexamfetamine who presented after a syncopal episode witnessed by his wife at home. He was found to be in ventricular fibrillation and subsequently had an extensive workup with cardiac magnetic resonance imaging (MRI), ischemic evaluation, and electrophysiology, which were unrevealing. He then received an automatic implantable cardiac defibrillator and was incidentally found to have hereditary hemochromatosis on outpatient follow-up. His polypharmacy may have potentially led to catecholamine release, leading to ventricular arrhythmia.

## Introduction

Recreational drug use is a significant public health concern in various countries. It is well understood that usage of psychedelics/hallucinogens such as lysergic acid diethylamide (LSD), ecstasy, phencyclidine (PCP), and psilocybin-containing mushrooms has increased significantly over the last few decades, particularly in adolescents and young adults [1]. Studies show that psilocybin-containing mushrooms, aside from their known hallucinogenic properties, have a particularly low side-effect profile and toxicity, with most side effects being benign and self-limiting [2]. It is necessary to further investigate such recreational drugs for their severe clinical effects, with some studies and case reports describing rare complications such as hyperthermia, seizures, coma, and acute kidney injury [3,4]. Hereditary hemochromatosis is a disorder characterized by iron deposition in tissues with organ damage. Cirrhosis, diabetes mellitus, arthritis, cardiomyopathy, and hypogonadotrophic hypogonadism are considered classical features. Cardiac disease secondary to hemochromatosis is less prevalent compared to hepatic manifestations; however, it typically manifests as a conduction abnormality, congestive cardiac failure, and cardiac dysrhythmias [5]. Here, we present the case of an adult male who presented with cardiac arrest associated with psilocybin use and was incidentally found on genetic testing to have hereditary hemochromatosis.

## Case presentation

A 48-year-old male with a past medical history of attention-deficit/hyperactivity disorder on lisdexamfetamine presented after a syncopal episode witnessed by his wife at home. Per his wife, the patient was in his normal state of health when he suddenly became unresponsive with "sudden shaking and seizure-like" activity described as "slow rhythmic movements with his left shoulder" mid-conversation while seated at home. The wife called emergency medical services (EMS) and began cardiopulmonary resuscitation with compressions. Upon arrival, EMS found the patient unresponsive, and an automated external defibrillator revealed the patient to be in ventricular tachycardia. He was immediately defibrillated, which resulted in a narrow complex supraventricular tachycardia with the return of spontaneous circulation. He was then mechanically cardioverted, and normal sinus rhythm was achieved. He was intubated out in the field and sedated with propofol. On the scene, EMS noted that mushrooms were found on site, which the wife identified as psilocybin-containing mushrooms. The wife otherwise denied any other medical history or medications for the patient, but his family history was significant for multiple first-degree relatives with sudden cardiac deaths.

**Figure 1:**
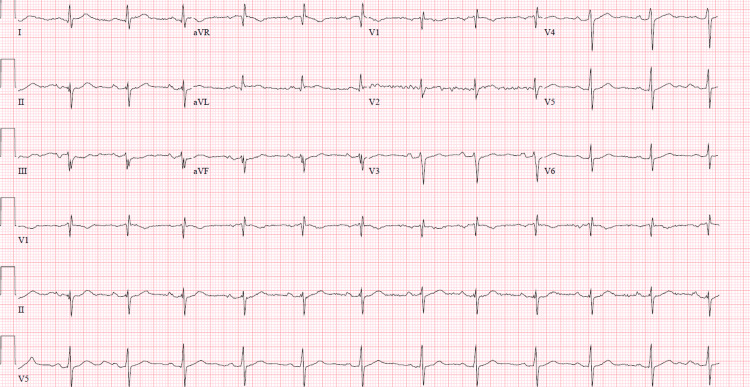
EKG showing right bundle branch block with left anterior fascicular block as well as fragmented QRS

On arrival at the emergency department, vitals were unremarkable, and the patient was hemodynamically stable while intubated. The physical exam was unrevealing. Labs were only remarkable for a Troponin I high sensitivity assay of 140 ng/L with a peak of 1489.6 ng/L, but there were no gross metabolic abnormalities such as electrolyte disturbances or acidosis nor signs of organ dysfunction such as elevated creatinine and transaminitis. HbA1c was 5.2%, and the lipid panel showed an LDL of 122 mg/dL. Urine toxicology was only remarkable for tetrahydrocannabinol (THC). The electrocardiogram (EKG) showed a right bundle branch block with the left axis deviation consistent with the left anterior fascicular block and a fragmented QRS notable in the inferior leads (Figure 1). The chest X-ray and CT of the head were unremarkable.

The patient was accepted into the coronary care unit and started on the post-cardiac arrest therapeutic hypothermia protocol. A transthoracic echocardiogram (TTE) showed an ejection fraction of approximately 65% with no evidence of segmental wall motion abnormalities, but there was evidence of left ventricular hypertrophy with concentric left ventricular remodeling (Video 1).

**Video 1:** Transthoracic echo with no evidence of segmental wall motion abnormalities

Given the preserved ejection fraction, no evidence of acute ischemia on EKG, and the lack of segmental wall motion abnormalities, suspicion for the acute coronary syndrome was low. The patient was not initiated on the acute coronary syndrome protocol with higher suspicion of drug-induced ventricular arrhythmia. A video electroencephalogram was also performed, which showed no epileptiform pattern or evidence of seizure activity, and the patient was successfully extubated on day two of hospitalization. 

**Figure 2:**
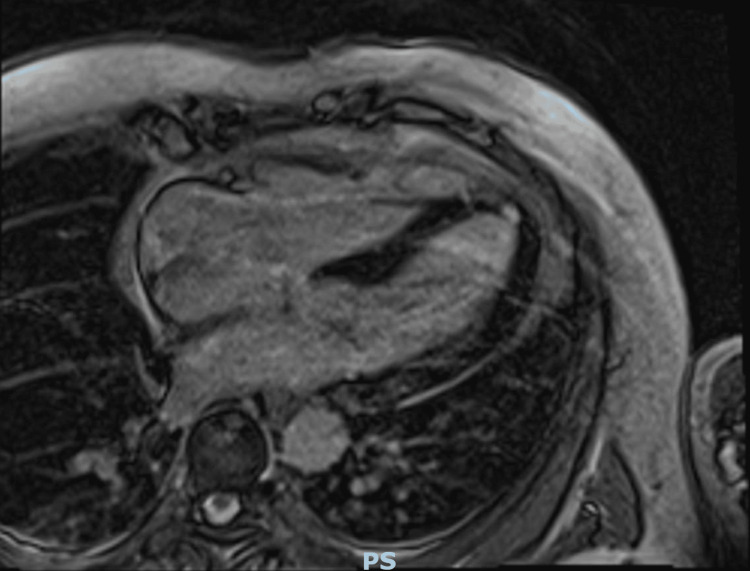
Cardiac MRI showing no evidence of late gadolinium enhancement

To definitively rule out coronary artery disease as an etiology of ventricular fibrillation, a coronary angiogram was obtained, only showing mild non-obstructive coronary artery disease. Given conduction disorders as well as fractionated QRS, cardiac MRI (CMRI) was also obtained to assess for fibrotic tissue as well as an infiltrative disease to explain the etiology of the patient’s ventricular fibrillation. Imaging showed no late gadolinium enhancement to suggest ischemia, scar tissue, or fibrosis and showed only evidence of left ventricular hypertrophy similar to the previously performed TTE (Figure 2).

The patient then underwent an electrophysiology study to assess for underlying sustained arrhythmias that could devolve into ventricular fibrillation. The patient’s baseline QTc was 405ms. He was started on an epinephrine infusion at 0.025 mcg/kg/min, which was incrementally increased to a maximum dose of 0.2 mcg/kg/min, but the QTc was still 424ms. A catheter was then placed in the right atrium, coronary sinus, right ventricular apex, and his positions. Pacing in the right ventricle revealed concentric ventriculoatrial conduction, but the conduction was decremental. Burst pacing from the right atrium induced atrial fibrillation that did not devolve into a rapid ventricular response with no changes in the QRS morphology, and the arrhythmia terminated spontaneously. A single atrial extra stimulus was delivered from the proximal coronary sinus with no evidence of dual atrioventricular nodal physiology. The patient then received a procainamide infusion with no evidence of Brugada syndrome or changes in his Bundle-Ventricular interval on continuous EKG monitoring. Finally, ventricular stimulation was performed at two separate locations in the right ventricle with no arrhythmias induced. An automatic implantable cardioverter-defibrillator (AICD) was placed for secondary prevention, and the patient was discharged with instructions to discontinue his lisdexamfetamine and avoid both marijuana and psilocybin use.

At the one-month follow-up, the patient was seen as an outpatient by cardiology, with AICD device interrogation showing no evidence of ventricular arrhythmia. The patient also followed up with outpatient genetic testing, which revealed a biallelic HFE C282Y mutation consistent with hereditary hemochromatosis. Outpatient iron studies showed a total iron level of 275 ug/dL and a ferritin level of 3068 ng/mL. He was referred to hematology with the recommendation for weekly phlebotomy.

## Discussion

Here, we present a case of cardiac arrest associated with psilocybin use. Incidentally, the patient was found to have hereditary hemochromatosis at outpatient follow-up. Psilocybin is a hallucinogenic compound that is rapidly converted to psilocin after ingestion, acting as a serotonin receptor agonist. Common side effects clinically include agitation, confusion, mydriasis, nausea, tachycardia, and hypertension [3]. Recreational use of psilocybin typically revolves around its hallucinogenic effects, which have previously been reported as relatively safe as most fatal outcomes were deemed to be a direct result of agitation and confusion rather than direct toxicity [6]. 

Currently, psilocybin has been studied for its antidepressant properties with relatively benign side effects, and cardiac side effects are typically limited to transient increases in heart rate and blood pressure [7]. A recent randomized control trial compared escitalopram to psilocybin, showing similar efficacy for depression, with psilocybin having a milder side effect profile [8]. However, recent literature reveals case reports showing associations between psilocybin use and more serious adverse events, including acute kidney injury [4] and notably cardiac arrest, as seen in our patient. 

Two case reports have reported psilocybin as a potential trigger of Takotsubo Cardiomyopathy (TTC). The first case involved a 17-year-old male presenting with chest pain and dyspnea. Initial workup was remarkable for widespread ST elevations, right bundle branch block on EKG, and elevated cardiac enzymes [9]. Coronary angiography was negative for the obstructive disease, with left ventriculography showing a regional wall motion abnormality in the apical segment and an EF of approximately 40%. CMRI confirmed the same findings. LV function appeared to have normalized with TTE, and a repeat CMRI was performed six days later. The second case involved a 59-year-old male with anxiety and depression who presented with visual hallucinations and respiratory distress after ingestion of Psilocybe semilanceata [10]. Initial EKG was remarkable for left bundle branch block, progressive anterolateral T-wave inversion, and prolonged QT interval. The patient had elevated troponin levels and a chest X-ray that was concerning for pulmonary edema. Echocardiography was significant for a dilated left ventricular end-systolic cavity with akinesia of the entire apex, mid-cavity septal, and lateral walls. Coronary angiography was negative for the obstructive disease, and CMRI performed five days later showed partial improvement in LV systolic function with extensive myocardial edema throughout the LV. On follow-up, repeat CMRI showed residual hypokinesis of the apical septum and improved myocardial edema. Repeat EKG and TTE were unremarkable with the resolution of his previous findings. 

Psilocybin as a potential trigger of TTC remains heavily understudied as a potential complication of recreational use. In both cases, there were no identified cardiac risk factors before the presentation and a negative workup for coronary obstruction, suggesting recent psilocybin ingestion as the most likely etiology of TTC. Psilocybin-induced cardiovascular toxicity likely involves catecholamine pathways, specifically acting on adrenergic, dopaminergic, and serotonergic receptors. The adrenergic response from the activation of these pathways is theorized to play a role in the development of TTC. Visual hallucinations may also serve as an emotional trigger for TTC and contribute to its development. Notably, TTC with emotional triggers as its primary etiology has been shown to have a better prognosis compared to TTC with physical stress triggers [10]. Further studies are necessary to delineate the precise mechanisms behind psilocybin-induced TTC to better inform prognosis and identify potential future treatment options. 

It is well established that a wide range of recreational drugs can increase the signaling mechanisms of the sympathetic nervous system (SNS) and hypothalamic-pituitary-adrenal axis [11]. Although drugs of abuse are mainly thought to involve modulation of the release of monoaminergic neurotransmitters, such as serotonin and dopamine, one study describes the increase of norepinephrine and epinephrine as a partial mechanism for the intoxicating effects of such drugs [11]. Norepinephrine and epinephrine released from the SNS have stimulatory effects involving an elevation in heart rate and blood pressure, rapid breathing, increased body temperature and diaphoresis, and pupillary dilation. Psilocybin may be converted to catecholamines via a chemical reaction that involves the release of the benzene ring from the rest of the molecule, thus forming dopamine, which can be easily converted into norepinephrine and epinephrine [11]. THC also seems to activate the sympathetic nervous system [12]. The increase in SNS signaling caused by both psilocybin and THC may have contributed to the development of cardiac dysfunction in this patient.

Hereditary hemochromatosis is an autosomal recessive disorder disrupting iron regulation, leading to multiorgan dysfunction through systematic iron deposition. Classically, hereditary hemochromatosis causes restrictive cardiomyopathy with impaired diastolic dysfunction and cardiac hypertrophy at first before progressing into dilatative cardiomyopathy with overt manifestations of heart failure as well as conduction disorders and arrhythmias [13,14]. Our patient only had findings of cardiac hypertrophy with conduction abnormalities on the EKG. However, while supraventricular arrhythmias seem to be associated with primary hemochromatosis, ventricular arrhythmias are not [14].

Our case stands out in that our patient, who did not have cardiac manifestations of late-stage primary hemochromatosis, went into ventricular fibrillation with normal electrophysiology studies and no evidence of TTC or restrictive cardiomyopathy. He also did not have any hepatic manifestations. A multifactorial etiology due to underlying hereditary hemochromatosis as well as polypharmacy from lisdexamfetamine, tetrahydrocannabinol, and psilocybin may have played a role in his cardiac arrest.

## Conclusions

This case illustrates the potential danger of psilocybin use as a potential trigger for ventricular arrhythmias leading to cardiac arrest. Previous case reports show TTC as a potential consequence of psilocybin use. Our case differs in that the patient did not have TTC pathology on TTE or CMRI and had unremarkable electrophysiology studies. His underlying hereditary hemochromatosis most likely played a role, as well as polypharmacy consisting of agents that could lead to activation of the sympathetic nervous system and catecholamine release.
